# Dr. Drake, on the Cure of Consumption

**Published:** 1799-12

**Authors:** Nathan Drake

**Affiliations:** Hadleigh, Suffolk


### *To the Editors of the Medical and Phyſical Journal.*

Gentlemen,

As few objects, I ſhould imagine, can be of greater importance, either to medical men or to the world at large, than the eſtabliſhment of the anti-phthiſical powers of the fox-glove, and as it is only by repeated and unequivocal inſtances of its ſalutary effects that ſo active an article of the Materia Medica can be brought into general and permanent uſe, I lately tranſmitted to you a caſe, in addition to thoſe of Marris and Grimes, in which it had effected a perfect cure, and ſome in which it had relieved and rendered ſtationary the more prominent ſymptoms of the diſeaſe.

The experience I have now had, indeed, thoroughly convinces me that the digitalis will be found adequate to the cure of ſeveral caſes of tubercular conſumption, though advanced into the ſecond ſtage; that it will, for a time, ſuſpend the progreſs of the ſymptoms in almoſt every inſtance; and that in catarrh, in hæmoptoe, and in vomica, it will, in ſome meaſure, approach to what is commonly implied by the term ſpecific; a word, however, which ſhould be baniſhed from medicine, when aſſuming the appellation of a ſcience, it being obvious to every one acquainted with the phænomena and mutability of vital action, that no ſubſtance whatever can be ſuppoſed to operate on the human ſyſtem in one uniform and undeviating manner.

Some apprehenſions are nevertheleſs entertained, I perceive, that injury may accrue to medicine from the reports already publiſhed in favour of this plant. Your Journal for Auguſt contains an ingenious communication from Dr. Maclean, of Sudbury; in which, however, notwithſtanding his own teſtimony to the good effects of the digitalis in conſumption, he concludes a paragraph, and commences another in the following words: "I ſee," ſays he, "the moſt ſerious evils begin "already to reſult from its not anſwering the high expectations that "have been raiſed."—"Others begin to loſe their confidence in it "from ſimilar failures; whereas, had it been brought forward with its "true character ſtamped upon it, this would not be the caſe."

Now, in the "Contributions to Medical and Phyſical Knowledge," the digitalis is related to have been given with a view to cure *tubercular*

*confumption*; and if, by experience, it has been found beneficial in catarrh, hæmoptoe, and fpafmodic afthma, does it follow that, fhould it fail in the former complaint, it muft naturally fall into difrepute in the latter difeafes? Surely Dr. MACLEAN fhould have treated with contempt thofe who are weak enough to think and argue in this manner. It has for feveral years been given in pulmonary hæmorrhage with effect, and certainly will continue to be, with the intelligent, whatever may be the refult of its trial in phthifis. I am happy, however, to fay, that the fuccefs which has hitherto attended the exhibition of the digitalis in phthifis has been confiderable; feveral patients in its confirmed ftate have been cured by this remedy, almoft all have been relieved; life has ever been protracted by it; and when death has taken place, whilft the fyftem was under its influence, it has been free from pain or ftruggle; my expectations have been anfwered, and Dr. FOWLER, I underftand, from further trials, is fixed in his former favourable opinion.

I may alfo with confidence affirm, that no evil of any magnitude can arife from the ufe of digitalis in tubercular confumption, if properly exhibited; and that he who fhall haften to employ it early in the difeafe, will, in proportion to his promptitude, be a benefactor to mankind. Every other medicine, when this complaint is once formed, has been found, by the experience of ages, worthlefs; many of them frivolous in the extreme; and to have recourfe to the old inefficient plan, when the fox-glove is within reach, is wilfully to give up the patient to certain diffolution.

That there are many difeafes which affume the form of phthifis, which may be removed by the cuftomary methods, and in which the digitalis would be unneceffarily employed, I am well aware; I am likewife well convinced that mifchief may, and will, probably, be produced by an injudicious and indifcriminate ufe of this active plant; but to what is this to be attributed? not fo much to any thing deleterious in the fox-glove, as to the ignorance and inattention of the practitioner. A fimilar fate has awaited almoft every valuable and active agent in the materia medica; but, as Dr. MACLEAN has very juftly obferved, at the clofe of his remarks on the effects of fox-glove, "It is unfair to argue "of the ufe of any fubftance from the abufe of it."

The moft unpleafant fymptoms confequent on a liberal and long-continued ufe of this medicine, are vertigo, naufea, and ficknefs; to remove or mitigate thefe, therefore, without diminifhing at the fame time, the the effect upon the circulation, becomes an object of primary importance. A few drops of laudanum with each dose of the tincture of digitalis will sometimes prevent the rejection of the latter from the stomach, though I have not found it very effectual either in removing the sensation of languor, or the affection of the head. In producing these beneficial purposes, the vegetable acid has, with me, proved much more serviceable. I was induced to make use of it from recollecting its utility in preventing similar effects, in certain constitutions, from the administration of opium. Thirty or forty drops of laudanum in a table-spoonful or two of pure lemon juice, will generally procure refreshing sleep without either heating the body, or being followed the next morning by nausea or vertigo. In one case, where the stomach and head were soon disordered by even a small dose of the digitalis, the lemon-juice produced an immediate good effect, removing both the sickness and vertigo, and enabling me to throw in a larger quantity of the tincture with ease and safety. This, however, being the only instance in which I have employed it, future experience must decide with regard to its general utility. To another patient, with the same view, I likewise gave eight or ten drops of the diluted nitrous acid with every dose of tincture, but could not perceive that it moderated its action on the stomach. Though I have never made the experiment, I am inclined to think that the tepid bath, where not much debility is present, would assist greatly in promoting the retardation of the circulating fluids, and might supersede the necessity of using very large doses of the digitalis for this purpose. Water, when heated to 94 or 96 of Farenheit's thermometer, will frequently, and in the course of a very short time too, reduce the pulse ten, twenty, or sometimes even thirty strokes in the minute. Now, after the system has felt the influence of the digitalis, and in about an hour after taking a small dose of it, might not the tepid bath, thus assisted, produce such a depression of the pulse as could only be expected from a full dose of the tincture, or decoction. It would be acquired too, most probably, without either sickness or vertigo; but whether it would be equally permanent or not, remains to be decided. It is evident, also, that a tepid bath, morning and evening, can only be resorted to in incipient consumption, and where no colliquative perspiration has occurred.

My usual vehicle for the tincture of fox-glove has been the infusion of quassia, and, except in the case of Mr. Boutill, (the first of those described in my last communication) I have never seen it produce *ex-*

*cessive*

*ceffive* languor. In four inftances I have reduced the pulfe to 40, 44, 50, and 56, with very little affection either of the head or ftomach. With fome patients, however, (fee firft and fecond cafes of my laft) thefe have proved fo violent as to preclude the profecution of the medicine. I have endeavoured, but hitherto in vain, to afcertain certain phænomena in the form, fyftem and habits of the patient, which might lead the phyfician immediately to difcriminate with accuracy thofe conftitutions which are fufceptible of the full and falutary influence of the digitalis from thofe which are not. It is a curious fubject, and would carry the inquirer into a wide phyfiological field; the vaft importance of fuch a difcovery, however, fhould ftimulate to further refearch.

As to the preparation of fox-glove beft adapted to enfure the requifite effect in confumption, I muft, from attentive experience, give a decided preference to the tincture, when thrown into comparifonwith the powder. Not only a larger quantity of the former can be *gradually* introduced into the fyftem with fafety, but it likewife acts more powerfully and uniformly in retarding the circulation. A material difference, too, may be obferved in their mode of operation; for, whilft the powder ufually increafes, and changes in point of colour the evacuation of urine, I have never, but in one inftance, and that in a very flight degree, perceived a fimilar effect from the ufe of the tincture. Of the infufion or decoction, from my own obfervation, I can fay nothing, not having yet applied either in this complaint; their efficacy in anafarca, however, I know to be great, and the fuccefs of Dr. Fowler has fufficiently proved that they are not lefs efficient in phthifis.

I perfectly agree with Dr. Maclean, in thinking it a matter of the firft confequence, that an uniform and ftandard tincture fhould be eftablifhed by authority. I am alfo of opinion, that the *faturated* tincture, when duly prepared, will beft anfwer this intention. I have lately made ufe of five ounces of proof fpirit to one ounce of the leaves coarfely powdered, without any dilution of the colour, or diminution of ftrength or tafte. Beyond this, however, it can offer no claim to the term *faturated*, as it becomes not only paler in colour, but weaker in flavour, and a larger quantity is required to produce a given depreffion of the pulfe. Provided the digitalis be good, and the fpirit accurately proof, the tincture, in the proportion of five to one, will be fully faturated, and will be, therefore, always of an uniform ftrength. Thus made, it appears of a deep brown colour approaching toward black, yet tinging paper paper or linen, when immerſed in it, of a beautiful green hue ; it has a ſtrong narcotic odour, and leaves a nauſeous and very bitter flavour on the palate. I have more than once, I am ſorry to ſay, met with the tincture ſo improperly made, that it has been not much deeper in colour than brandy, and poſſeſſing little ſmell or taſte. The *ſaturated* tincture ſhould be cloſely confined from the air, and kept in a mild temperature; for, when expoſed to any great degree of cold, it loſes its tranſparency, and depoſits a part of the digitalis in the form of a very fine powder. Another circumſtance to be attended to in exhibiting the tincture is, that it be always dropped from the ſame phial, an ounce or two-ounce one, for inſtance, as the drop will be larger or ſmaller in proportion to the ſize of the rim of the phial.

With regard to the plant itſelf, I can aſſure Dr. Maclean, he is deceived in ſuppoſing it to acquire greater vigour from cultivation in a garden. Nineteen times out of twenty he will find the reverſe to be the event. The digitalis delights in an elevated, a light and gravelly ſoil ; on a fat and denſe mould, in low and damp ſituations, and where ſtrata of chalk abound, it always degenerates, becomes dwarfiſh, and of a paler green. Dr. Maclean's houſe at Sudbury, is certainly ſituated low, not far from the river, and in the immediate neighbourhood of large chalk-beds; on ſuch a ſoil, it is evident, none of the habitudes of the plant can be gratified ; and by the uſual modes of improving a garden, the ground will be rendered ſtill leſs fit for promoting its growth and ſtrength. I have repeatedly ſeen it cheriſhed in ſhrubberies for the beauty of its flowers, but never yet ſaw it attain the heighth and ſtrength of the wild plant. In laſt July, I gathered at Bergholt, about ſix miles from this town, in company with Mr. Travis, the ſurgeon of the above place, and Mr. Bunn, of Hadleigh, a very large quantity of the digitalis. It grows in profuſion in this diſtrict, from the adaptation of the ſoil ; and we nearly filled a common ſized cart with plants gathered from the hedges of one field. A vaſt number of theſe were from five to ſix feet in heighth, and with ſtems and leaves of proportional ſize ; the latter frequently of a dark duſky green, approximating to brown, exhaling a ſtrong ſmell ſomething ſimilar to tobacco, and poſſeſſing an intenſely nauſeous bitter taſte. The leaves of a brighter green, were ſmaller, and had *comparatively* little odour or flavour ; the dark and duſky leaves too, were uſually found upon the talleſt and ſtrongeſt plants. I have been the more particular in mentioning theſe circumſtances, with a view to prevent, unleſs where the ſoil and ſituation are congenial to the fox-glove, its its cultivation on garden ground, and, therefore, its confequent degeneracy.

The inferences which I have drawn from fome well-known facts in phyfiology, and to which I am indebted for the ufe of digitalis in confumption, appear to me to have been mifunderftood by Dr. Maclean, and to have been objected to upon infufficient grounds. In the prefent ftate of medical fcience, no man, who has duly cultivated it, and who wifhes to diftinguifh his practice from empiricifm, can or ought to exhibit a new and powerful agent without founding fuch exhibition on the deductions of fcience and analogy. What I have delivered in the "Medical Contributions," on the probable action of the digitalis, is the mere chain of analogical reafoning, which induced me, when called to Mr. Marris, firft to prefcribe the fox-glove in confumption. When I reflect on the event of this cafe, and of fome others which have fince occurred, I may be allowed, I think, from motives of pure benevolence, to look back upon this affociation of idea with mingled emotions of pleafure and gratitude.

"There are fome modern practitioners," obferves Dr. Darwin, "who declaim againft medical theory in general, not confidering that "to think is to theorize; and that no one can direct a method of cure "to a perfon labouring under difeafe without thinking, that is, without "theorizing; and happy, therefore, is the patient, whofe phyfician "poffeffes the beft theory."*

Impreffed with the truth of this obfervation, I fhall attempt to prove, and it will be no difficult tafk, I think, that what has been termed "a "theory totally inadmiffible," is but, in fhort, a ftatement of facts, drawn from broad experience, and that the only part which can merit the appellation of theory, is a mere analogical deduction arifing from thefe facts. In order to render this more clear, I fhall ftate, in numerical order, the different data. 1. Pus is a fecreted fluid, the confequence of certain difeafed motions of the extremities of the blood-veffels. 2. Hectic fever arifes only from the matter of an open ulcer. 3. What is termed laudable pus, when fecluded from the air, is neither capable of creating fever, nor, except by its gravity, can it irritate the parts on which it refts. 4. When pus is expofed to atmofpheric air, it

* Zoonomia, vol. i. preface, page 2.

it rapidly attracts oxygen, and an acid of a peculiar kind is generated.
5. Hectic fever is the effect of the absorption of aerated matter.

Now, should it be made to appear, that there is just ground for thinking these affirmations to be matters of fact, no one will, probably, deny that the curative processes are legitimately deduced, namely, either to promote absorption so rapidly from the surface of the diseased parts, that the pus shall be taken up as soon as secreted, and, consequently, its combination with oxygen prevented; or so powerfully to retard the motion of the heart and circulating fluids, that the irritating and morbid action of the extremities of the blood-vessels, and, therefore, secretion, as its immediate effect, should be considerably diminished, or altogether suspended.

As Dr. M. has admitted the probable truth of the first and fourth of these data, it will not be expected that I should occupy any portion of my paper in an attempt to prove what he himself has not chosen to controvert. It may, however, be of use to observe, that ample information may be obtained on these topics from Brugman,* Everard Home,† and Darwin.‡ With regard to the second *datum,* namely, that hectic fever arises only from the matter of an open ulcer, Dr. Maclean affirms, that "daily observation contradicts it, and that in the early "stages of consumption it is well known, that the hectic fever is often "clearly and distinctly marked, without any increased expectoration, and "when the tubercles are still in their infancy, and, consequently, before "they have suppurated." And in a subsequent page, "that hectic "arises from collections of matter in different parts of the body, and "more especially from vomicæ in the lungs themselves, without any "communication with the external air."

That fever frequently arises during the formation of tubercles in the lungs, or from indurations of the lymphatic glands of the mesentery, and sometimes from encysted and unexposed matter, either owing to an inflammation of the walls of the vomica, or to the distension and gravity of the secreted fluid, is a circumstance of daily observation; but that this fever is one and the same with what is termed hectic fever, and which arises from aerated pus, the experience of the most celebrated practitioners will, I think, positively contradict. "The hectic now de-"scribed,"

* Inaugural Dissertation, Leyden, 1787.

† Dissertation on the Properties of Pus, 1788.

‡ Zoonomia, vol. i. and ii.

"fcribed," fays CULLEN, "as accompanying a purulent ftate of the "lungs, is, perhaps, the cafe in which it moft frequently appears; but "I have never feen it in any cafe when there was not evidently, or "when I had not ground to fuppofe, there was a permanent purulency "or ulceration in fome external or internal part. Indeed, it appears "to me to be always the effect of an acrimony abforbed from ab- "fceffes or ulcers, although it is not equally the effect of every fort of "acrimony; for the fcorbutic and cancerous kinds often fubfift long in "the body without producing a hectic."*

So effentially different did thefe fevers appear to Dr. DARWIN, both in their caufes and fymptoms, that he has given them diftinct appellations, and arranged them as diftinct fpecies, under the terms FEBRIS A PURE CLAUSO, and FEBRIS A PURE AERATO. "The former is dif- "tinguifhed," he obferves, "from the fever from aerated matter in "open ulcers, becaufe there are feldom any night fweats or colliquative "diarrhœa in this, as in the latter. The pulfe is alfo harder, and re- "quires occafional venæfection, and cathartics, to abate the inflamma- "tory fever, which is liable to increafe again every three or four days; "till at length, unlefs the matter has an exit, it deftroys the patient. "In this fever, the matter, not having been expofed to the air, has not "acquired oxygenation, in which a new acid is produced, which acts "like contagion on the conftitution, inducing fever fits, called hectic "fever, which terminate with fweats or diarrhœa; whereas, the matter "in the clofed abfcefs is either not abforbed, or does not fo affect the "circulation as to produce diurnal or hectic fever fits; but the ftimulus "of the abfcefs excites fo much fenfation as to induce perpetual py- "rexia, or inflammatory fever, without fuch marked remiffions. Ne- "verthelefs, there fometimes is no fever produced, when the matter is "lodged in a part of little fenfibility."† On the FEBRIS A PURE AERATO, he fays, "a great collection of matter often continues a "long time, and is fometimes totally abforbed, even from venereal "buboes, without producing any diforder in the arterial fyftem. At "length, if the ulcer has been opened, fo that any part of it has "been expofed to the air for but one day, a hectic fever is produced. "Whence the utility arifes of opening large abfceffes by fetons, as, in "that cafe, little or no hectic fever is induced; becaufe the matter is "fqueezed out by the fide of the fpongy threads of cotton, and little or "no

* Firft Lines, vol. ii. p. 861.

† Zoonomia, vol. ii. p. 282.

"no air is admitted; or by tapping the abfcefs with a trochar. In this "fever, the pulfe is about 120 in a minute, and its accefs is generally "in an evening, and fometimes about noon alfo, with fweats or purging "towards morning, or urine with pus-like fediment; and the patients "bear this fever better than any other with fo quick a pulfe."* In fpeaking, too, of the FEBRIS MESENTERICA arifing from matter formed in the mefenteric glands, he remarks, that the patient is deftroyed by the continuance of fimple pyrexia, or inflammatory fever; for, "as "the matter is not expofed to the air, no hectic fever, properly fo "called, is induced."†

I have had feveral opportunities of attending clofely to the fever arifing from mefenteric induration, both previous to and long after open ulceration had taken place among the mufcles of the back, and I have obferved an effential difference in the nature of the fever accompanying thefe oppofite ftates. One melancholy cafe was under my daily obfervation in this place for more than four years. The patient was about fixteen, when I firft faw him; was of a very fcrofulous habit; and was fuppofed to have laboured many years under mefenteric indurations, and with all the fymptoms likewife of tubercles in the lungs. His friends informed me, that feveral years ago, but long after his indifpofition commenced, a confiderable tumor appeared among the mufcles of the back and loins, and which terminated in fuppuration, and a large open ulcer: He was then attacked with all the fymptoms of hectic fever, and it was the opinion of his medical attendants, that he could not furvive long. Contrary to their expectations, however, the ulcer healed; the hectic difappeared; and he arrived in Hadleigh, I was told, precifely in the fituation he had been in, previous to the fuppurative procefs; that is, he had difficulty of breathing upon motion; an almoft perpetual teazing cough, but no expectoration; an emaciated and contracted form; little thirft; great appetite; and an uniform, flight, and continued fever. In this ftate he remained for about three years, when two confiderable tumours were again formed among the mufcles of the back; and in fpite of every preventive mean, fuppuration largely followed, and with great pain. On confultation with Dr. GIBBONS, of this place, it was thought advifeable, as the matter pointed outwardly, and occafioned much irritation, to evacuate the pus of both abfceffes; they were accordingly opened by his surgeon, Mr. BUNN, and hectic fever with strong exacerbations, and all its train of symptoms, shortly followed, and terminated in his death about a twelvemonth after; the ulcers, notwithstanding every effort to promote inflammation, absorption, and consequent adhesion, never healing. This remarkable case always appeared to me, and long before I had seen the Zoonomia of Dr. DARWIN, as a decisive proof, that the fever following open ulceration, was essentially different in its nature from that which existed previous to it.

As to the third affirmation, namely, that what is termed laudable pus, when secluded from the air, is neither capable of creating fever, nor, except by its gravity, can it irritate the parts on which it rests; it should be observed, that pus, when first secreted, has been found by very accurate experiments to be a transparent fluid, and to assume the globular and opaque form only after it has rested for some time on the surface of the sore; when secluded from the air, fifteen or twenty-five minutes are required to effect this change, which seems, in a great measure, the consequence of the absorption of the inner parts. It is perfectly mild, and free from any corrosive properties, and following the laws of all secreted fluids, never affects, whilst in its original pure state, the surface which produced it. "It is always in harmony," says Mr. "HOME, with the parts which form it, having no power of irritating "them, even when the surrounding parts are affected by it.—This seems "peculiar to secretions, and arises from the parts themselves not being "susceptible of irritation from their own matter."* The absorption of pus in this its native state, produces no effect whatever upon the animal œconomy, whence the utility of stimulating the absorbents to take it up previous to any vitiation, or any exposure to the air. That fever, however, frequently arises from the formation of pure pus, is undoubtedly true; it is always preceded by inflammation, and sometimes by great pain; by its gravity also, and distension among neighbouring parts of great sensibility, the same effect will often be produced. This pyrexia, however, the result of the physical, not chemical properties of pus, may be accurately distinguished from hectic fever. Pus also is susceptible of many vitiated states, though secluded from the air; but the fever thence arising, and which is termed by DARWIN, FEBRIS A PURE CONTAGIOSO, presents no one characteristic of hectic.

In noticing the fifth and last division, namely, that hectic fever is the

* On the properties of pus, pages 59 and 61.

the effect of the abforption of aerated matter, I muft refer to what has been already written under the fecond, what is there faid being equally applicable to the prefent fubject. It is of great importance, however, to add, that Dr. PRIESTLEY has by experiment difcovered, that oxygen fo greedily unites with animal fubftances, that it will pafs through a moift bladder to combine with them; that BRUGMAN in his Thefis, likewife by experiment, found that pus when expofed to the air in a moderate heat becomes acidified; and that Dr. BEDDOES, from clofe attention to a variety of curious and minute phænomena, with great probability infers, that the ftate of body predifpofing to phthisis, and which continues during the progrefs of the difeafe, is a ftate of hyper-oxygenation.*

Before I difmifs the fubject, however, I cannot avoid commenting upon two remaining objections of Dr. MACLEAN, and which appear to be founded on miftaken and partial views. "If this were the cafe," obferved Dr. M. (viz. that hectic fever is the effect of the abforption of aerated matter) "the thick bland matter fecreted from every wound "or ulcer, when expofed to a ftream of air, would become an ichorous "poifon, and be productive of the effects mentioned by Dr. DRAKE; "but that this does not in reality happen, daily obfervation fufficiently evinces." Now it occurs very unfortunately for this objection, that the recent improvement in the treatment of ulcers and abfceffes, is precifely that of avoiding expofure to the air, and the confequent vitiation of the pus; for it has been the refult of univerfal experience, I believe, that the action of the air on ulcers, in a greater or lefs degree, always produces acrimony of the purulent matter. "It is remarkable", fays DARWIN, "that matter produced by fuppuration, "will lie concealed in the body many weeks, or even months, without "producing hectic fever; but as foon as the wound is opened, fo as "to admit air to the furface of the ulcer, a hectic fever fupervenes, "even in a very few hours.—Hence, when ulcers are to be healed, it "is neceffary, carefully to exclude the air from them. Hence we have "one caufe which prevents pulmonary ulcers from healing, which is, "their being perpetually expofed to the air."† Mr. ABERNETHY, who has met with great fuccefs in the treatment of lumbar abfcefs, by gradually evacuating the pus with a trochar, and thence excluding the admiffion of air, attributes the hectic fever which follows, when air is incautioufly

* Obfervations on Confumption, 93. † Zoonomia, vol. i. p. 299, 300

incautioufly admitted, to mere inflammation of the walls of the abfcefs; but it remains to be proved, that fimple inflammation has ever yet given rife to hectic fever. Were this the cafe, inflammation of the abdomen and of the tunica vaginalis teftis from the admiffion of air, and of the coats of a vein after bleeding, would produce hectic fever; it fhould be recollected, that inflammation is the fole caufe of the fecretion of healthy pus; and when air is admitted to the fides of an abfcefs, it can only act upon their furface as a fimple ftimulus, occafioning frefh inflammation, and a further fecretion of pure pus; thefe are, however, of themfelves frequently fufficient to exhauft a patient already debilitated; but as hectic fever and a vitiated purulency are almoft always the confequence of the expofure of an abfcefs or ulcer to the atmofphere, it would clearly feem to follow,. that hectic fever is the effect of the abforption of pus, after it has received from the air fome noxious material.

The laft objection which Dr. Maclean has brought forward to the doctrine of the abforption of matter in phthisis, is, that ulcers are never healed by abforption, and that the furgeon fhould not endeavour to promote it. "Good pus," the Dr. remarks, "he would look for "as a neceffary confequence; nor would he, under any circumftances, "endeavour to promote its abforption." This objection appears to me the more extraordinary, as it is the language of medical men, that no ulcer can be healed without abforption; nay, this procefs is abfolutely neceffary to the production of what is called laudable pus; for when firft fecreted, it is tranfparent and comparatively thin, and only acquires its opacity and confiftence from the abforption of the more fluid parts; and even this laudable pus muft difappear, though at firft neceffary to granulation, before the ulcer can heal. The following paffages place this matter in a very clear light. "The art of healing ulcers," fays the author of Zoonomia, "confifts in producing a ten-"dency to abforption in the wound greater than the depofition. Thus "when an ill-conditioned ulcer feparates a copious and thin difcharge, "by the ufe of any ftimulus, as of falts of lead, &c. externally appli-"ed, the difcharge becomes diminifhed in quantity, and it becomes "thicker, as the thinner parts are firft abforbed. But nothing fo much "contributes to increafe the abforption in a wound, as covering the "whole limb above the fore with a bandage; by this artificial tight-"nefs of the fkin, the arterial pulfations act with double their ufual "power in promoting the afcending current of the fluid in the valvular "lymphatics. Internally, the abforption from ulcers fhould be promot-

"ed

" ed first by evacuation, then by opium, bark, mercury, steel." Again, " no ulcer can heal, unless the absorption from it is as great as " the deposition in it;" that is, ulcers cannot heal when " the secre- " tion of matter in them continues to be greater than the absorption " of it."*

From the whole of what has been now said, the two curative indications in phthisis, namely, either to promote the absorption of pus previous to its aeration, or so to retard the circulation as altogether to preclude the purulent secretion, will appear, I should hope, both obviously and accurately deduced. As to the digitalis, it has been found, by repeated experiments, to effect in curing consumption, either one or both these purposes, and even in incipient tubercular phthisis before ulceration has taken place; I suspect the great benefit received from the employment of this medicine, may arise from the absorption of the tubercles themselves. " In what I have judged imminent consumption," " says Dr. Beddoes, " the digitalis has produced the most salutary ef- " fects in at least as many cases as it has failed. The fatal consequences " of hæmoptoe have been prevented; and either the symptoms associated " with tubercles removed, or (what I am disposed to believe, but time " alone can fully decide) absorption of the tubercles themselves has ta- " ken place. An assertion like this, is, I am well aware, liable to be " controverted; and it is incapable of absolute proof, since it is impossi- " ble to take tubercles out of a diseased thorax and exhibit them. " The probability indeed of their existence is not always equal; but of " the nature of the disorder, in most cases, I feel confident—so exactly " similar were the appearances to those which I had so often observed " before ulceration of the lungs in other cases; and it is scarce possible " I should have misjudged in many of the instances: of this, not only " the perfect identity of symptoms, but the coinciding opinion of more " than one medical man, afforded security."†

I have thus, in as brief a manner as possible, endeavoured to prove, that the opinions and deductions brought forward in the paper on phthisis, in the Medical Contributions, were not built upon slight grounds, but were the result of a survey of many physiological facts, which, when thrown together, appeared to me satisfactorily to account for both the salutary effects of digitalis, and the state of the system in phthisis. I wish

* Zoonomia, vol. i. 410; vol. ii. 730.

† Essay on Consumption, 2d edition, page 303.

wish it, however, to be recollected, that no obliquity or subserviency can attach to practice in consumption, in consequence of what has been delivered; the two facts with regard to digitalis, viz. its powers of promoting absorption, and its dominion over the heart and arteries, stand prominent and insulated from any set of opinions; and the cases too, in which its salutary agency has been recorded, can admit of no modification. The attempt to throw light upon the nature of an obscure but destructive disease, and to account for the effects of the most powerful agent yet employed in removing this disease, can only be productive of benefit: should it fail, it may have the merit of eliciting further and more successful inquiry; but should it approximate toward the truth, incalculable may be the utility conferred upon mankind.

I am, GENTLEMEN,

With great respect,

Yours, &c. &c.

*Hadleigh, Suffolk, Oct.* 28, 1799. NATHAN DRAKE.

